# A comprehensive neural simulation of slow-wave sleep and highly responsive wakefulness dynamics

**DOI:** 10.3389/fncom.2022.1058957

**Published:** 2023-01-13

**Authors:** Jennifer S. Goldman, Lionel Kusch, David Aquilue, Bahar Hazal Yalçınkaya, Damien Depannemaecker, Kevin Ancourt, Trang-Anh E. Nghiem, Viktor Jirsa, Alain Destexhe

**Affiliations:** ^1^CNRS, Institute of Neuroscience (NeuroPSI), Paris-Saclay University, Saclay, France; ^2^Institut de Neurosciences des Systèmes, Aix-Marseille University, INSERM, Marseille, France; ^3^Laboratoire de Physique, Ecole Normale Supérieure, Université PSL, CNRS, Sorbonne Université, Université de Paris, Paris, France

**Keywords:** neural simulation, mean-field model, spontaneous activity, evoked responses, wake, synchronous, slow-wave sleep, human brain

## Abstract

Hallmarks of neural dynamics during healthy human brain states span spatial scales from neuromodulators acting on microscopic ion channels to macroscopic changes in communication between brain regions. Developing a scale-integrated understanding of neural dynamics has therefore remained challenging. Here, we perform the integration across scales using mean-field modeling of Adaptive Exponential (AdEx) neurons, explicitly incorporating intrinsic properties of excitatory and inhibitory neurons. The model was run using The Virtual Brain (TVB) simulator, and is open-access in EBRAINS. We report that when AdEx mean-field neural populations are connected *via* structural tracts defined by the human connectome, macroscopic dynamics resembling human brain activity emerge. Importantly, the model can qualitatively and quantitatively account for properties of empirically observed spontaneous and stimulus-evoked dynamics in space, time, phase, and frequency domains. Large-scale properties of cortical dynamics are shown to emerge from both microscopic-scale adaptation that control transitions between wake-like to sleep-like activity, and the organization of the human structural connectome; together, they shape the spatial extent of synchrony and phase coherence across brain regions consistent with the propagation of sleep-like spontaneous traveling waves at intermediate scales. Remarkably, the model also reproduces brain-wide, enhanced responsiveness and capacity to encode information particularly during wake-like states, as quantified using the perturbational complexity index. The model was run using The Virtual Brain (TVB) simulator, and is open-access in EBRAINS. This approach not only provides a scale-integrated understanding of brain states and their underlying mechanisms, but also open access tools to investigate brain responsiveness, toward producing a more unified, formal understanding of experimental data from conscious and unconscious states, as well as their associated pathologies.

## 1. Introduction

Brain activity is marked by complex spontaneous dynamics, particularly during conscious states when the brain is most responsive to stimuli. Though changes in spontaneous and evoked dynamics have been unambiguously empirically observed in relation to changes in brain state, their multi-scale nature has notoriously occluded a formal understanding.

Spanning from macroscopic dynamics supporting communication between brain regions to microscopic, molecular mechanisms modulating ion channels, hallmarks of consciousness have been observed across spatial scales. At the whole-brain level, conscious states are marked by complex spontaneous collective neural dynamics (Niedermeyer and Lopes da Silva, 2005; El Boustani and Destexhe, 2010) and more sustained, reliable, and complex responses to stimuli (Massimini et al., 2005; Casali et al., 2013; D'Andola et al., 2018; Dasilva et al., 2021). At the microscopic level, neuromodulation is enhanced in conscious, active states, leading to microscopic changes in cellular kinetics (McCormick, 1992). Yet, a challenging multi-scale problem still resides in comprehending how changes in the complexity of global spontaneous dynamics and whole brain responsiveness may specifically relate to microscopic neuromodulatory processes to enable neural coding during active states. Here, using mean-field models of conductance-based, Adaptive Exponential (AdEx) integrate-and-fire neurons with spike-frequency adaptation developed recently (Zerlaut et al., 2018; Capone et al., 2019; di Volo et al., 2019), constrained by human anatomy and empirically informed by local circuit parameters, we report the natural emergence of global dynamics mimicking different human brain states.

To connect microscales (neurons) to macroscales (whole brain), this work relies on previous advances at mesoscales (neural populations). The first step was modeling biologically-relevant activity states in networks of spiking neurons. Based on experimental recordings, we used the Adaptive Exponential (AdEx) integrate and fire model to simulate two main cell types identifiable in extracellular recordings of human brain (Peyrache et al., 2012): regular-spiking (RS) excitatory and fast-spiking (FS) inhibitory cells. AdEx networks were constrained by biophysical representations of synaptic conductances, which allowed the model to be compared to conductance measurements done in awake animals (Zerlaut et al., 2018) (for experiments, see Steriade et al., 2001; Rudolph et al., 2007). In such configurations, AdEx networks reproduce states observed *in vivo* (Destexhe, 2009; Jercog et al., 2017; Zerlaut and Destexhe, 2017; Zerlaut et al., 2018; Nghiem et al., 2020), notably asynchronous irregular (AI) states found experimentally in awake states, and synchronous slow waves as in deep sleep (Destexhe et al., 1999; Steriade et al., 2001; Steriade, 2003). From AdEx networks, mean-field models were derived to take into account second order statistics of AdEx networks interacting through conductance-based synapses. We used a Master Equation formalism (El Boustani and Destexhe, 2009), modified to include adaptation (di Volo et al., 2019).

In this manuscript, we present evidence that mean-field descriptions of biophysically informed estimates of neuron networks produce macroscopic dynamics capturing essential characteristics of human wake and sleep states—due to variation in spike-frequency adaptation—when coupled by the human connectome with tract-specific delays. First, we show that simulated microscopic changes in membrane currents directly lead to the emergence of globally asynchronous vs. synchronous dynamics exhibiting distinct signatures in the frequency domain, as well as changes in inter-regional correlation structure and phase-locking, mimicking aspects of spontaneous human brain dynamics. The spatial extent of synchrony and phase relations across brain regions was observed to be an emergent property of both microscopic-scale adaptation changes and the organization of the human connectome, which allow for enhanced phase coherence at intermediate, cross-region, but not whole-brain scales in sleep-like states, consistent with the propagation of traveling waves. Further, we report enhanced brain-scale responsiveness to stimulation in simulations of asynchronous, fluctuation-driven compared to synchronous, phase-locked regimes, consistent with empirical data from conscious vs. unconscious brain states. Together, the data suggest that the TVB-AdEx model represents a scale-integrated neuroinformatics framework capable of recapitulating known features associated with human brain states as well as elucidating relationships between space-time scales in brain activity. Due to its reliance on anatomical data non-invasively available from humans, this model may further facilitate subject-specific modeling of human brain states in health and disease, including restful and active waking states, as well as sleep, anesthesia, and coma to aid future advances in personalized medicine.

## 2. Results

We begin by showing essential properties of the components forming the TVB-AdEx model. Next, we describe the integration of AdEx mean-fields into The Virutal Brain (TVB) simulator of EBRAINS, making the models and analyzes openly available to facilitate replication and extension of the results. The results presented here indicate that the TVB-AdEx whole human brain model captures fundamental aspects of synchronous and asynchronous brain states, both spontaneously and in response to perturbation.

### 2.1. Components of TVB-AdEx models

**Figure 1:**
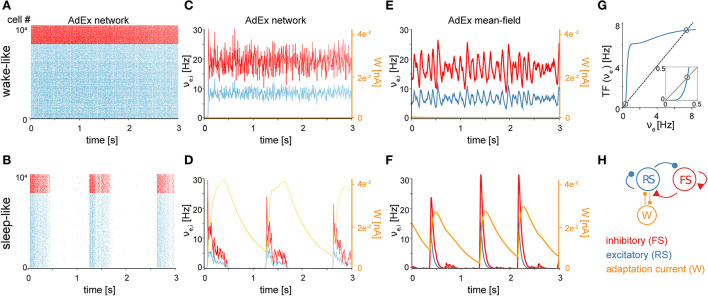
Asynchronous and synchronous dynamics produced by networks of microscopic AdEx neurons and their mesoscopic approximations. Raster plots **(A, B)** and mean firing rates **(C–F)** from networks comprised of excitatory RS (blue) and inhibitory FS (red) AdEx neurons displaying asynchronous **(A)** vs. synchronous states **(B)** as in Capone et al. (2019) and di Volo et al. (2019). The two simulated states, mimicking wake and sleep neural dynamics, differ only in the spike-frequency adaptation current *b*_*e*_ provided to the model (*b*_*e*_ = 0 *pA* in the asynchronous state and 60 *pA* in the synchronous state), known to be regulated by neuromodulation *in vivo* (McCormick, 1992). **(C, D)** Time variation of mean firing rates (ν_*e,i*_) and adaptation current (*W*_*e*_) corresponding to networks shown in **(A, B)**. Asynchronous **(E)** and synchronous **(F)** firing rate dynamics produced using a mean-field model of AdEx networks implemented in The Virtual Brain (TVB). **(G)** Input-output firing rate relations are given by the mean-field model transfer function (TF). Mean output firing rates of excitatory (blue) neurons as a function of mean excitatory input. The dashed black trace is the identity line. Fixed points of the system (gray circles) occur where the input-output relation intersects with the identity at the positions marked by circles (see Methods for equations). Note that two fixed points are apparent, one at high firing rates and one at low firing rates. The inset is an enlargement of the low-input, low-output region, highlighting the presence of the low-firing fixed point. During asynchronous, wake-like states, firing rates fluctuate around the higher fixed point. During sleep-like states, spike-frequency adaptation builds up as excitatory neurons fire at the high-rate fixed point, eventually destabilizing the high-rate fixed point and causing the system to transition to the near-zero rate fixed point. While the neurons are near-silent, adaptation decays through time, allowing noise fluctuations to entrain a transition back to the high-rate fixed point. **(H)** Schematic of the simulated network.

The first component of the TVB-AdEx model is at the cellular level, and consists of networks of integrate-and-fire adaptive exponential (AdEx) neurons. As shown in previous studies (Destexhe, 2009; Zerlaut et al., 2018; di Volo et al., 2019), networks of AdEx neurons with adaptation can display asynchronous, irregular (AI) states, as well as synchronous, regular slow-wave dynamics that alternate between periods of high activity (Up) and periods of near silence (Down). The necessary mechanistic ingredients needed to obtain both dynamical regimes include leak conductance and conductance-based synaptic inputs. Each neuron's input is comprised by the firing rates of synaptically connected neurons, weighted by synaptic strengths, as well as stochastic noise (hereafter called “drive”; see Materials and Methods), related biologically to miniature postsynaptic currents. AdEx neurons have the ability to integrate synaptic inputs and fire action potentials, followed by a refractory period (Brette and Gerstner, 2005). AdEx networks with conductance-based synapses can capture features offered by more detailed and computationally expensive models, including AI states and slow-wave dynamics. Figure 1 shows an example of such AI states (Figure 1A) and Up-Down dynamics (Figure 1B) simulated by the same AdEx network, changing only the level of spike-frequency adaptation current (parameter *b* in the equations, see Material and Methods). In AI states, the firing of individual units remains irregular, but sustained (Figure 1A), whereas in slow-wave states the dynamics alternate between depolarized Up states with asynchronous dynamics and hyperpolarized Down states of near silence (Figure 1B). As such, changes in spike-frequency adaptation lead to differences in cellular kinetics between sleep and wake states. Biologically, spike-frequency adaptation is suppressed by enhanced concentrations of neuromodulators such as acetylcholine during active, conscious brain states that tends to close K^+^ leak channels, resulting in sustained depolarization of neurons (McCormick, 1992) which promotes the emergence of asynchronous, irregular (AI) action potential firing and fluctuation-driven regimes associated with waking states. In contrast, low levels of neuromodulation during unconscious brain states leave leak K^+^ channels open, leading to waves of synchronous depolarisation and hyperpolarization due to the buildup and decay of spike-frequency adaptation, accounting for the emergence of slow-wave dynamics as observed in previous modeling work (Jercog et al., 2017; Nghiem et al., 2020).

The second component of the TVB-AdEx model is a mean-field equation derived from spiking-neuron network simulations, capturing the typical dynamics of a neuron in response to inputs and hence able to describe the mean behavior of a neuronal population, Zerlaut et al. (2018) and di Volo et al. (2019) using a Master-Equation formalism (El Boustani and Destexhe, 2009). This formalism allows one to derive mean fields from conductance-based integrate-and-fire models. It has been shown that—using numerical fits of the transfer function (Zerlaut et al., 2016), an analytical expression for the relationship between a neuron's input and output rates - one can describe complex neuronal models, such as AdEx neurons, and even Hodgkin-Huxley type biophysical models (Carlu et al., 2020). In Figures 1C, D, excitatory and inhibitory firing rates are compared between mean-field simulations using the Master Equation formalism and spiking neural network simulations (time binned population spike counts divided by time bin length *T* = 0.5 ms). The average adaptation value is also shown for this network (Figures 1C, D, orange curves). These population variables are suitably captured by the mean-field model including adaptation (Capone et al., 2019; di Volo et al., 2019). This mean-field model can exhibit both AI (Figure 1E) and Up-Down dynamics (Figure 1F). Like in the AdEx spiking network model, the transition between two states can be obtained by changing the adaptation parameter called *b* in both cases (di Volo et al., 2019). With no adaptation, the dynamics are fluctuation-driven around a fixed point exhibiting nonzero firing rates. With adaptation, as the neurons self-inhibit due to adaptation, the nonzero rate fixed point is progressively destabilized by adaptation buildup, driving the dynamics back to the near-zero firing rate fixed point until adaptation wears off and noise drives the system back to the vicinity of the higher-rate fixed point. Thus, with adaptation, the system displays noise-driven alternation between the two fixed-points to generate slow waves (Figure 1G). The regimes are achieved using the mean-field model, which describes excitatory (RS) and inhibitory (FS) population firing rates as well as the mean adaptation level of excitatory populations (Figure 1H).

### 2.2. Integration of AdEx mean-field models in TVB

We have used the simulation engines of the Human Brain Project's (HBP's) EBRAINS neuroscience research infrastructure (https://ebrains.eu and The Virtual Brain https://ebrains.eu/service/the-virtual-brain) to make access as wide as possible. Replication of the TVB-AdEx findings can be done here, with a free EBRAINS account, and users can clone the repositories to further test or extend the present capacities. The models can also be downloaded from Github at https://gitlab.ebrains.eu/kancourt/tvb-adex-showcase3-git to run locally.

**Figure 2:**
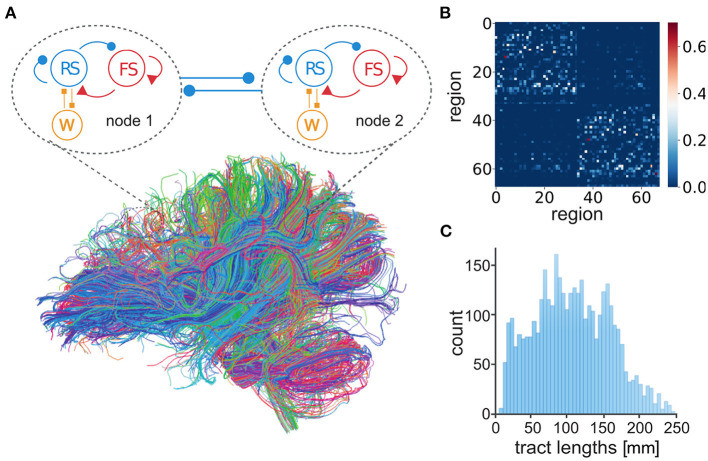
Connection of AdEx mean-field models in The Virtual Brain. **(A)** Each mean-field model consists of two populations, excitatory RS (blue) and inhibitory FS (red) neurons (as in Figure 1H), taking into account spike-frequency adaptation for excitatory neurons (*W*, orange). Mean-field models represent the mesoscopic scale, here comprising each of 68 defined regions of cerebral cortex. Brain regions are connected by excitatory tracts (thick blue lines) following structural connectomes (Schirner et al., 2015). **(B)** Number of fibers connecting brain regions in tractography data, divided by the sum of the gray matter volume of regions in anatomical MRI, is used to define connectivity weights between nodes. **(C)** The distribution of tract lengths in tractography data informs delays between TVB-AdEx model nodes.

Here, the connection of mean-field models was defined by human tractography data (https://zenodo.org/record/4263723, Berlin subjects/QL_20120814) from the Berlin empirical data processing pipeline (Schirner et al., 2015) (Figure 2A). A parcellation of 68 regions was used to place localized mean-field models, with long-range excitatory connections (Figure 2B) and delays (Figure 2C) defined by tract length and weight estimates in human diffusion tensor imaging (DTI) data (Sanz-Leon et al., 2015). Now it becomes possible to simulate brain-scale networks using AdEx-based mean-field models in TVB, hence the name “TVB-AdEx” model.

### 2.3. Spontaneous dynamics of large-scale networks

**Figure 3:**
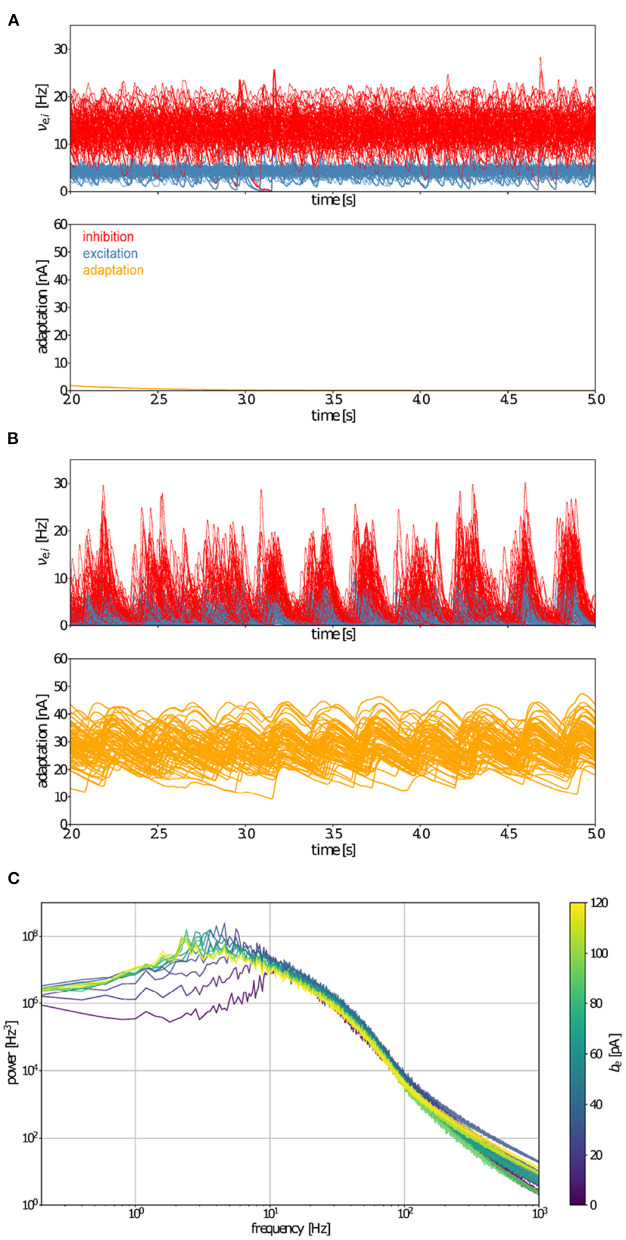
Whole-brain-scale simulations of connected AdEx mean-field models produce activity mimicking wake- and sleep-like states. Time variation of firing rates (ν_*e,i*_, top) and adaptation currents (*W*_*e*_, bottom) in simulated wake- **(A)** and sleep-like **(B)** states for each of the model nodes representing 68 brain regions. When adaptation (*b*_*e*_) equals 0 pA, the activity of model nodes is asynchronous **(A)**, whereas the inclusion of adaptation (*b*_*e*_ = 60 *pA*) leads to the emergence of synchrony between brain regions **(B)**. **(C)** Fourier power spectra of signals produced by the TVB-AdEx in synchronous (sleep-like) and asynchronous (wake-like) states for different values of *b*_*e*_. Note that maximal power in the sleep-like condition falls in the delta range (1–4 Hz), while it occurs near 10 *Hz*, in the alpha range for low adaptation, wake-like states.

Having coupled AdEx mean-field models that capture average microscopic characteristics of neural activity, we sought to ascertain if hallmarks of brain-scale (macroscopic) spontaneous activity resembling human brain states were reproduced, as well as whether increases in adaptation strength account for transitions between wake-like and sleep-like macroscopic dynamics. Characterizing temporal hallmarks of simulated neural activity (Figure 3), we find that asynchronous, wake-like dynamics across nodes are recovered in the absence (*b* = 0 *pA*, Figure 3A), but not in the presence (*b* = 60 *pA*, Figure 3B) of adaptation. Power spectral analysis reveals a peak in the delta range (0.5 − 5 Hz) for the high-adaptation condition (Figure 3C) consistent with empirical data from deeply sleeping individuals. Further, the power spectrum in the low-adaptation condition shows a maximum near 10 *Hz* (alpha range), consistent with empirically observed dynamics during resting wakefulness (Figure 3C). Therefore, changes in a simulated microscopic process (spike-frequency adaption) influence spectral features of macroscopic brain states, with low-adaptation regimes resembling waking states and high-adaptation regimes reminiscent of slow-wave sleep.

**Figure 4:**
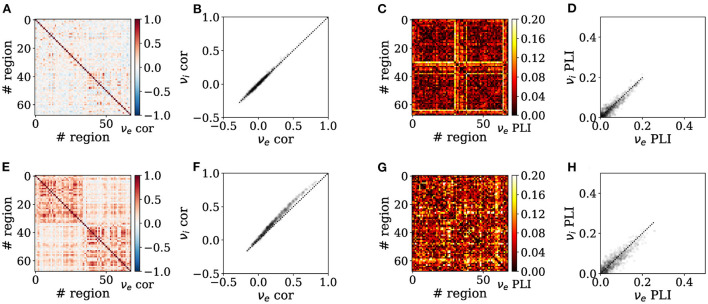
Emergence of enhanced spontaneous synchrony between brain regions in sleep-like simulations. Functional connectivity is assessed in wake-like **(A–D)** and deep sleep-like **(E–H)** states, by assessing Pearson correlation **(A, B, E, F)** and Phase-Lag Index (PLI) **(C, D, G, H)**. Heatmaps show correlations between brain regions in terms of excitatory firing rates **(A, C, E, G)**, whereas scatter plots of show relationships between inhibitory vs. excitatory firing rate correlations **(B, D, F, H)** where the dotted trace is the identity line. Inter-region correlations are increased across regions in sleep-like states **(E)** as compared to wake-like states **(A)**, consistent with increased synchrony across brain regions in empirical brain imaging studies (M/EEG). Correlations across nodes are significantly larger between inhibitory firing rates than between excitatory firing rates in sleep-like dynamics [**(F)**; Independent Student's *t*-test, *t* = −8.5, *p* = 2.8*e* − 17], but not during wake-like regimes [**(B)**; *t* = −0.9, *p* = 0.35]. The PLI is consistently larger in sleep-like dynamics **(G)**, unlike in wake-like dynamics where the PLI is diminished **(C)**. Likewise, the PLI of excitatory vs inhibitory populations is significantly different during sleep-like [**(H)**; Independent Student's *t*-test, *t* = 5, *p* = 4.6*e* − 7], but less so in wake-like [**(D)**; *t* = 4.2, *p* = 1.8*e* − 5] states, altogether possibly suggesting a previously unidentified role of inhibition in the emergence of long-range synchrony in sleep-like activity.

Increasing adaptation can also tune the spatial correlation structure of neural activity across brain states. Indeed, as shown in Figure 4, Pearson correlations across nodes are enhanced in the presence of adaptation, consistent with asynchronous dynamics seen during wakefulness vs. synchronous slow waves seen during deep sleep (Figures 4A, E). We also observe the correlation matrix is structured into two clusters corresponding to the two hemispheres in the slow-wave condition (*b* = 60 pA, Figure 4E). In addition, increased adaptation strength also causes the emergence of significantly larger correlations between inhibitory than excitatory firing rates across nodes during sleep-like dynamics (Figures 4B, F). This reveals that microscopic variation in adaptation strength alone can account for empirical reports of increased correlations between inhibitory neurons across long distances and even different cortical regions specifically for inhibitory (Peyrache et al., 2012; Le Van Quyen et al., 2016; Olcese et al., 2016). This is due to different effects of adaptation on excitatory regular-spiking neurons and inhibitory fast-spiking neurons, key to reproducing empirical dynamics in unconscious states (Jercog et al., 2017; Nghiem et al., 2020). Moreover, the Phase Lag Index (PLI) is increased during sleep-like dynamics (Figures 4C, G), suggesting systematic phase relations between nodes consistent with traveling slow waves empirically observed during spontaneous unconscious dynamics (Destexhe et al., 1999; Steriade, 2003). Such phase relations, evidenced by a significantly larger PLI, are more pronounced for inhibitory than excitatory neurons in sleep-like dynamics, reminiscent of the key role of inhibitory neurons in organizing the emergence of synchronous dynamics during sleep (Nghiem et al., 2018).

**Figure 5:**
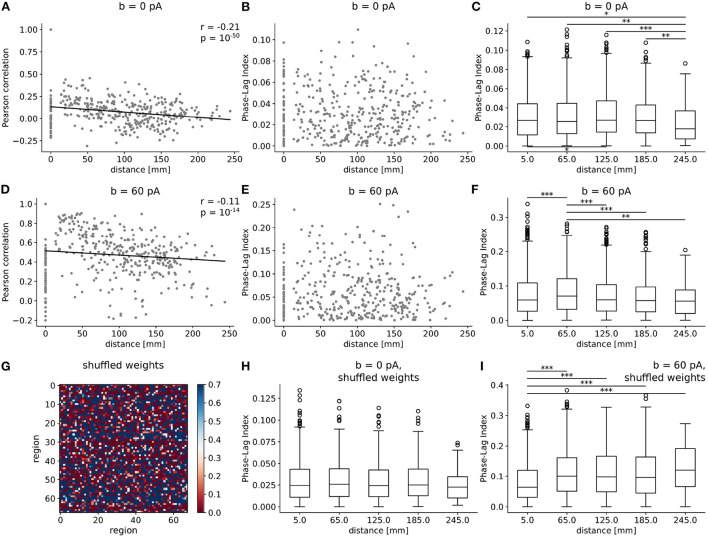
Spatial extent of synchrony and phase coherence across regions emerge from adaptation strength and connectome structure. **(A–F)** Pearson correlation **(A, D)** and PLI **(B, C, E, F)** between regions as a function of distance, as scatter plots **(A, B, D, E)** and box plots **(C, F)** in the absence [*b* = 0 pA, (**A–C**)] and presence [*b* = 60 pA, **(D–F)**] of adaptation. In scatter plots, 400 points were subsampled randomly for graphical representation, and solid black lines represent linear fits. Correlations are observed to decrease with distance, in a steeper manner in the wake-like condition. In box plots, distances between regions are subdivided into five bins of equal size, and the mean distance between the two extremities of each bin is marked on the horizontal axis. The PLI is significantly enhanced, suggesting increased phase coherence, at an intermediate spatial scale in slow-wave dynamics. **(G)** Heatmap of connection weights in shuffled connectivity matrix, where the weights from one region to every other region are successively randomly permuted. **(H, I)** Boxplot of PLI as a function of distance for simulations with a shuffled connectome for *b* = 0 pA **(H)** and *b* = 60 pA **(I)** [compare to **(C, F)**]. The *, **, and *** symbols indicate the values of *p* < 0.05, *p* < 0.01, and *p* < 0.001 respectively.

Next, we investigate how the connectome's structure shapes the landscape of synchrony and phase coherence across brain regions, alongside adaptation. In particular, how do the Pearson correlation and PLI scale with spatial distance between nodes? In both *b* = 0 pA and *b* = 60 pA conditions (Figures 5A, D), the Pearson correlation between excitatory firing rates significantly decreases with Euclidean distance between regions, corresponding to tract-length-related delays in our model. A steeper negative slope is observed in the awake-like (Figure 5A) than in the slow-wave regime (Figure 5D), suggesting that the spatial extent of synchrony between regions is enhanced in the presence of high-adaptation, sleep-like dynamics.

In Figures 5B, C, E, F, we show a scatter plot and box plot of the Phase-Lag Index (PLI) as a function of distance between regions. In both *b* = 0 pA and *b* = 60 pA cases, significant differences are observed in the PLI across regions (Kruskal-Wallis test, ***p < 0.001), suggesting systematic phase relations consistent with the propagation of traveling waves are particularly predominant at intermediate scales. Specifically, in the slow-wave condition (*b* = 60 pA), we observe that the PLI between regions approximately 65 mm apart is significantly enhanced (Mann-Whitney U test, **p < 0.01) in comparison to the PLI at both shorter and longer distances.

Further, we test whether the predominance of slow waves at an intermediate spatial scale is an emergent property from the structure of the human connectome. To this purpose, we shuffle the connection weights successively for each region and every other region, to generate a connectome with the same distribution of connection weights between regions but retaining none of the graph structure of the empirical connectome (Figure 5G). The tract lengths are not modified. Repeating simulations with shuffled connectomes, we again compute the PLI as a function of distance. With shuffled connectomes, we find that the PLI no longer varies significantly as a function of distance in wake-like dynamics (Figure 5H). As well, the intermediate peak in PLI as a function of distance—denoting an intermediate spatial scale for traveling waves (Figure 5I)—is lost. These results suggest that the non-trivial organization of phase coherence phenomena across cortex is an emergent property of both high adaptation and the weighted graph structure of the human connectome.

**Figure 6:**
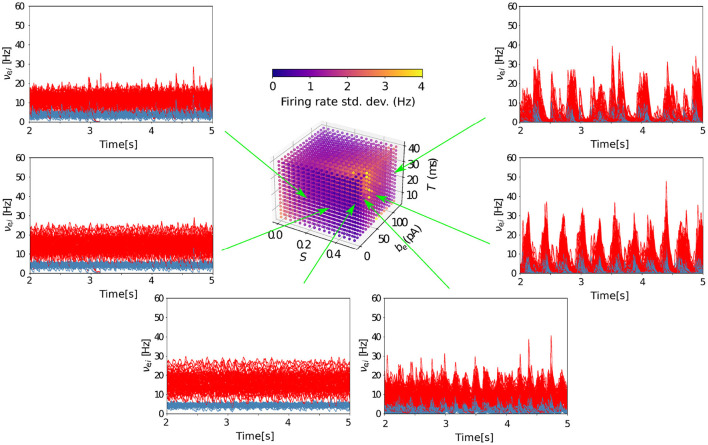
Grid search in parameter space for asynchronous and slow oscillatory states. The TVB-AdEx model was run for six different parameter combinations in the depolarized region (*E*_*L,i*_ = *E*_*L,e*_ = −64 mV) and the time traces of the inhibitory and excitatory populations of the 68 AdEx mean-field models are plotted during the last 3 s of the simulation. With *b*_*e*_ = 0 pA, for intermediate values of *S*, one can see how AI states appear, consistent with the low value of SD shown in the feature plot. For *S* = 0.5, one can see the transition between AI and UD states when increasing *b*_*e*_.

Finally, the transition between wake and sleep-like dynamics when changing the level of adaptation is robust for different combinations of parameters of the model (Figure 6). With a high-density scan of the parameter space (see Methods), we find that, for lower values of spike-frequency adaptation (*b* = 0 *pA*), AI states are present independently of the timescale (*T*) of the AdEx mean-field model network nodes and the coupling strength between nodes (*S*). Consequently, when increasing adaptation, there is a sharp transition from wake-like dynamics to slow-wave activity captured by a marked increase in firing rate standard deviation, again robustly across all values of *T* and *S* in the explored range.

### 2.4. Responsiveness to external stimulation

After reproducing features of spontaneous dynamics between brain states, we test the hypothesis that changing adaptation in the TVB-AdEx model can also explain differences in empirically observed stimulus-evoked brain responses, with stimuli encoded in more sustained, widespread, reliable, and complex patterns during conscious states (Massimini et al., 2005). To this end, a square wave of 0.1 *Hz*, matching the magnitude of stochastic drive, with 50 *ms* duration was input to the firing rates of the transfer function for excitatory populations in the right premotor cortex of the TVB-AdEx simulation during awake-like and slow wave sleep-like conditions, as in previously published empirical studies (Casali et al., 2013).

**Figure 7:**
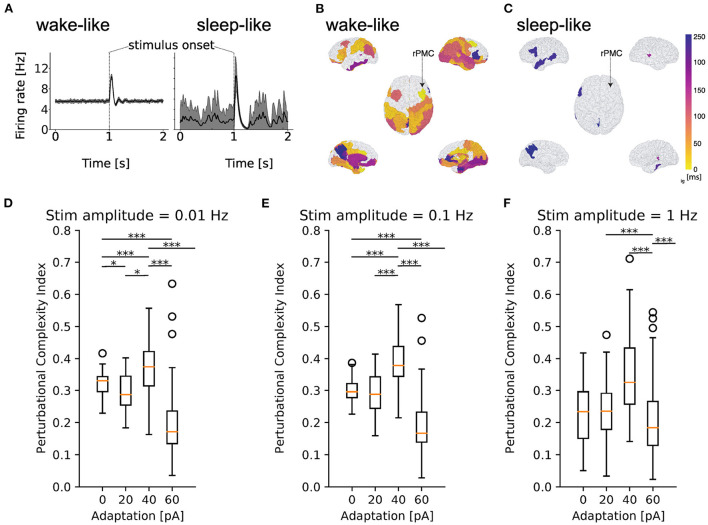
Increased responsiveness to perturbations in simulated wake-like compared to sleep-like states. Excitatory firing rate of a simulated brain region in time during wake-like **(A)** and sleep-like dynamics, in response to a stimulus. Black lines show the mean across 40 realizations, reminiscent of event-related potentials (ERPs), and gray shaded areas show the standard deviation from the mean across realizations. **(B, C)** Spatio-temporal propagation of responses to stimuli in wake-like **(B)** and sleep-like **(C)** states. Color plotted on the brain surface indicates earliest time at which each region becomes significantly different from its pre-stimulus baseline (see Methods), with earlier times in lighter colors. Regions in white do not present significant differences in firing rate in response to the stimulus. In wake-like states, stimulus responses recruit more brain regions and produce more spatially widespread and temporally sustained activity patterns, reminiscent of empirical observations. **(D–F)** Box plots of perturbational complexity index (PCI) measurements from 40 realizations of wake-like and sleep-like simulations with increasing stimulus amplitudes (panels left to right). Significant changes in the PCI are observed when the spike frequency adaptation (*b*_*e*_) is varied (one-way Kruskal-Wallis test; *p* < 0.05 for each group of adaptation values, *b*_*e*_ = 0, 20, 40, 60, for each stimulus value (0.01, 0.1, and 1 Hz). Results of *post-hoc* Conover test for multiple comparisons between values of *b*_*e*_ are shown in the figure, where *p < 0.05, **p < 0.01, and ***p < 0.001). In high-adaptation, sleep-like regimes, a sharp drop in PCI is observed, denoting more spatio-temporally complex responses in the Lempel-Ziv sense in wake-like compared to sleep-like states, consistent with experiments (Massimini et al., 2005; Casali et al., 2013).

Figure 7 illustrates the effect of perturbing the large-scale network defined by the TVB-AdEx models. The effect of an external stimulus is apparent for both deep sleep-like and wake-like states (Figure 7A). The average traces of the stimulated region are shown in black, and take into account the 40 realizations shown in gray. To examine the spread of activity following perturbations, the time at which the excitatory firing rate of each region becomes significantly different from the unstimulated baseline (prior to perturbation) is plotted using a color map showing earlier significant changes in brighter colors. Here, we find that responses are more widespread across time and space across brain regions in wake-like (Figure 7B) than sleep-like dynamics (Figure 7C), corresponding to experimental observations in response to Transcranial Magnetic Stimulation (TMS) (Massimini et al., 2005).

To better characterize the complexity of stimulus-evoked responses, the perturbational complexity index (PCI), used in previous experimental works involving TMS, was computed. The PCI is the ratio between Lempel-Ziv complexity, which captures the number of all possible different binary words that can be extracted from binarized responses to stimuli across time and regions, and the entropy of the binarized response that describes how often the response is above the pre-stimulus baseline (see Methods for binarization procedure). A low PCI value indicates a “simple” response to stimulus, while a high PCI value indicates more “complex” response, typically propagating more effectively to different brain areas (Casali et al., 2013). The models were perturbed with stimuli smaller (0.01 Hz) than the stochastic drive, comparable to the noise in amplitude (0.1 Hz), and larger than the drive (1.0 Hz) for simulations in which the value of spike frequency adaptation (*b*_*e*_) was varied between 0 and 60 pA. As shown in Figures 7D–F, computing the PCI from the TVB-AdEx model shows that PCI values are typically higher for lower-adaptation, wake-like regimes than for higher-adaptation, slow-wave sleep like regimes. In particular, a sharp drop in PCI values is observed between *b* = 40 pA and *b* = 60 pA, suggesting an abrupt transition between highly responsive asynchronous and less responsive slow-wave dynamics as adaptation increases. For each value of noise, a one-way ANOVA revealed significant differences between PCI distributions across *b*-values (*p* < 0.0001), with multiple comparisons highlighting that the PCI was significantly larger for wake-like than sleep-like conditions, in particular for lower-amplitude stimuli. The same behavior was observed when comparing awake subjects with subjects in slow-wave sleep (Massimini et al., 2005; Casali et al., 2013). One may note a sharp drop of the PCI after *b* = 40 pA, for which enhanced PCI is observed, reminiscent of a transition from conscious to unconscious response dynamics. As concentrations of neuromodulators such as acetylcholine are known to increase with attention and vigilance, b = 40 pA, which is at the higher-adaptation, lower-neuromodulation end of the spectrum of high-PCI states, could be reminiscent of states that lie between waking vigilance and deep sleep, such as resting wakefulness. Also note the wider distribution of PCI values in sleep-like simulations, suggesting more variable responses for each realization of the same stimulus and therefore less reliable stimulus encoding.

## 3. Discussion

In this paper, we demonstrated that biologically-informed scale-integrated mean-field models (di Volo et al., 2019) can be used to simulate large-scale brain networks using the TVB platform in EBRAINS. The coupled mean-field models comprising the TVB-AdEx are derived from networks of AdEx neurons and display whole-brain asynchronous and slow-wave dynamics when wired following white matter tracts from a human connectome. These results demonstrate the natural emergence of empirically observed patterns of macroscopic brain dynamics from simulated changes at microscopic scales from both microscopic adaptation changes and the structural organization of the human connectome. The TVB-AdEx integration in EBRAINS is also of interest as EBRAINS human brain atlas services will be able to provide a large degree of cytoarchitectural detail such as region-specific neurotransmitter densities and cell types and densities and thus add to the biological realism of these virtual brain models. The vertical integration across scales is provided by TVB-AdEx-type models, taking advantage of the Big Data in EBRAINS.

TVB-AdEx mean-field models constituting each node of the connectome are designed by construction to approximate the mean and covariance of the firing rate in spiking neural networks exhibiting stable dynamics in asynchronous irregular regimes (El Boustani and Destexhe, 2009). This model was extended to two neuronal populations, excitatory neurons with adaptation and inhibitory neurons (di Volo et al., 2019), but this extension has limitations. Importantly, the model is imprecise when adaptation varies within a range larger than described here [for adaptation values higher than 100 pA (di Volo et al., 2019)] and when fast synchronous dynamics like oscillations in the gamma range (between 40 and 80 Hz) (El Boustani and Destexhe, 2009), spindles, or ripples occur. This model is therefore likely not directly suitable for understanding the nuances associated with particular microscopic motifs comprised by transiently communicating assemblies that likely encode relevant neural information. The model is appropriate to the type of general dynamics presented here, wake-like AI and deep sleep-like slow-waves, describing large scale phenomena with relatively slow time scales. By smoothing microscopic details, we have built a computationally tractable bridge from microscopic to macroscopic scales, to elucidate how general dynamical phenomena relate to differences in neuronal interactions.

After integration in TVB, the resulting TVB-AdEx model displays a number of interesting features and several exciting perspectives for future work. A first result is the emergence of synchrony across brain regions in the presence of adaptation. In this case, the TVB-AdEx model displays synchronized slow waves with structured phase relations at a macroscopic, brain-wide level (Figure 3). This is consistent with the synchronized slow-wave dynamics observed during deep sleep in the brain (Destexhe et al., 1999; Steriade, 2003; Niedermeyer and Lopes da Silva, 2005). When the same model is set into the asychronous-irregular regime due to the loss of spike-frequency adaptation, as is the case in the presence of acetylcholine and other neuromodulators present in higher concentrations during waking states (Jones, 2003), the large-scale network displays a lower level of synchrony (Figure 3), consistent with asynchronous dynamics typically seen in awake and aroused states (Destexhe et al., 1999; Steriade, 2003; Niedermeyer and Lopes da Silva, 2005). These different levels of synchrony are therefore emergent properties in the large-scale network, induced by changes at the microscopic level. A detailed grid scan in parameter space established the robustness of this phenomenon (Figure 6).

A second main result is that evoked dynamics are also state-dependent. When the network displays synchronized Up- and Down-states, a stimulus typically evokes a high amplitude, simple response that remains local in space and time. When the model resides in the asynchronous regime, the same stimulus evokes responses that are weaker in amplitude, but that propagate in a more elaborate way through space and time. The PCI measure applied to these two states match the experimental observations (Massimini et al., 2005; Casali et al., 2013; D'Andola et al., 2018; Dasilva et al., 2021). Again, this is an emergent property of the large-scale network.

What are possible mechanisms for such differences? A previous study (Zerlaut and Destexhe, 2017) showed that in balanced networks, not all states are equal and that asynchronous states, despite their apparently noisy character, can display higher responsiveness and support propagation of stimuli. This enhanced responsiveness of AI states can be explained by the combined effects of depolarization, membrane potential fluctuations, and conductance state. It was proposed as a fundamental property to explain why the activity of the brain is systematically asynchronous in aroused states (Zerlaut and Destexhe, 2017). The present results are in full agreement with this mechanism, which manifests here in the asynchronous state as a propagation further in time and space, across many brain areas, associated with higher values of the PCI.

We believe that this work opens several perspectives. First, the enhanced propagation of perturbations during wake-like states could be used as a basis to explain why stimuli are perceived in asynchronous regimes, and what kind of modulation of the network activity could support phenomena such as attention and perception. Second, mean-field models can be set to also display pathological states, such as hyper-excitable or hypersynchronized states, and the TVB-AdEx model could be used to investigate seizure activity (Depannemaecker et al., 2021). Other features, such as neuronal heterogeneity, are also beginning to be included in mean-field models (di Volo and Destexhe, 2020), paving the way for enhancing biological realism in future versions of TVB-AdEX models.

In TVB, connectivity depends on the intermediate spatial resolution of coarse-graining. Here, the brain was parceled in 68 regions, with each mean-field representing a substantially large brain area. TVB allows such simple simulations using a few tens of nodes, taking into account the rough long-range connectivity according to the connectome resolved in tractography of human DTI. TVB can also simulate much finer-grained connectivity, by defining a larger number of nodes (usually on the order of hundreds to hundreds of thousands, approaching the resolution of cortical columns) (Spiegler and Jirsa, 2013). In such vertex-based simulations that shall follow the present work, local connectivity is determined by intracortical connections, whereas the white-matter connectome from DTI used here captures effects of longer-range cortico-cortical fibers.

Early stimulation studies in humans and in particular in rodents are pioneering the use of high-resolution simulations, demonstrating subtler influences of the connectome in scaffolding signal propagation through brain networks (Spiegler et al., 2016, 2020). In those studies, network nodes were equipped with generic neural mass dynamics, Andronov-Hopf-oscillators, which are theoretically appealing for their mathematical simplicity, but are limited with regard to biophysical interpretability of the results. The inclusion of high-resolution data from tracer studies in the Allen Institute was recently demonstrated in virtual mouse brain models to significantly increase the predictive power (Melozzi et al., 2019). As well, the inclusion of subject-specific, personalized connectomes in virtual brain models significantly outperforms generic simulations in predictive inter-individual variability (Melozzi et al., 2019; Hashemi et al., 2020). These studies point together to the importance of personalized brain network models in future clinical applications and affords novel methods supporting such goals (Falcon et al., 2016; Jirsa et al., 2017). The virtual brains in Spiegler et al. (2016, 2020) captured the emergence of well-known resting state networks known during spontaneous activity, but also functionally specific brain responses to stimulation of regions along the processing chains of sensory systems from periphery up to primary sensory cortical areas. The latter responses heavily relied on the Default Mode Network (DMN) and were suggestive of the DMN playing a mechanistic role between functional networks. But neither brain state dependence, nor biological interpretation of the neural mass model parameters was possible, as it requires the incorporation of biological complexity and integration across scales provided here by the here by the TVB-AdEx approach. Ongoing efforts in EBRAINS aim to enrich high-resolution brain models with detailed information on regionally-variant physiological features (neurotransmitters, receptor densities, cell types, and densities) to next build the Virtual Big Brain, a high-resolution multi-scale brain network, which will be continuously updated and available to the community. The drawback of such fine grained simulations is that they typically require large computing resources as provided by EBRAINS, while coarse grained TVB simulations, as presented here, can easily be run on a standard workstation. To summarize, for the sake of the initial release of the TVB-AdEx models, we offer a relatively coarse parcellation, which will become more refined and personalized in future work.

The TVB-AdEx models presented here are constructed by connecting conductance-based, mean-field models of biologically-informed populations of neurons by a human connectome (Sanz-Leon et al., 2015). While the results presented in the main figures are made on the backbone of a single example human connectome, and many of the reported emergent phenomena could be reproduced with other topologies of connectivity, it is imperative to note that the human connectome backbone of the TVB-AdEx model is interchangeable between human subjects, representing an opportunity to construct personalized models, digital twins (Evers and Salles, 2021; Petkoski and Jirsa, 2022), for any human subject for which diffusion tractography data are available. While a study of inter-individual variation is beyond the scope of this study, as a proof of principle, our data indicate differences in spectral features, particularly the power of low-frequency activity and power-law scaling, between personalized TVB-AdEx models made from two different healthy human subjects (Supplementary Figure S1). Indeed, a parameter scan using TVB-AdEx models derived from the subjects identifies different overlapping regions of parameter space in which transitions between sleep-like and wake-like activity between human subjects. Construction of next generation TVB-AdEx models with human connectomes for which biological and behavioral data are available, for example, from the Human Connectome Project cohorts, will allow researchers to use TVB-AdEx models methods introduced here to test predictions regarding inter-subject differences in brain states and their transitions. All code is openly available in the EU digital neuroscience platform EBRAINS (Schirner et al., 2022) and on Github to facilitate progress in personalized brain modeling (Falcon et al., 2016) of neural states and their transitions in health and disease.

It is interesting to note that the global properties used here to characterize neural dynamics across brain states—synchrony, frequency spectra, responsiveness, and PCI—all reflect neural correlates of consciousness (Skarda and Freeman, 1987; Tononi and Edelman, 1998; Sarasso et al., 2014; Koch et al., 2016). It has even been argued that asynchronous dynamics (so-called 'activated EEG') is so far one of the most “sensitive and reliable” neural correlates of consciousness (Koch et al., 2016). Though we have only presented results from stimulating one brain region in different brain states, in the interest of replicating experimental results (Casali et al., 2013), it is important to note that our approach offers the possibility to perturb any region within a given connectome to simulate the effects of various stimuli or tasks in a variety of states, as well as the dynamical consequences of transcranial stimulation in experimental and medical contexts. This further emphasizes the promise of the present modeling approach to understanding dynamics associated with conscious and non-conscious states, with broad potential applications in medicine and computation.

## 4. Materials and methods

Three types of models are used in this work: a network of spiking neurons, a mean-field model of this network, and a network of mean-field models implemented in The Virtual Brain (TVB). Here we describe these models successively.

### 4.1. Spiking network model

We considered networks of integrate-and-fire neuron models displaying spike-frequency adaptation, based on two previous papers (Destexhe, 2009; Zerlaut et al., 2018). We used the Adaptive Exponential (AdEx) integrate-and-fire model (Brette and Gerstner, 2005). We considered a population of *N* = 10^4^ neurons randomly connected with a connection probability of *p* = 5%. We considered excitatory and inhibitory neurons, with 20% inhibitory neurons. The AdEx model permits to define two cell types, “regular-spiking” (RS) excitatory cells, displaying spike-frequency adaptation, and “fast spiking” (FS) inhibitory cells, with no adaptation. The dynamics of these neurons is given by the following equations:

(1) $$\begin{array}{l} c_{m}\frac{dv_{k}}{dt}=g_{L}\left(E_{L}-v_{k}\right)+g_{L}\Delta e^{\frac{v_{k}-v_{thr}}{\Delta }}-w_{k}+I_{syn} \end{array}$$

(2) $$\begin{array}{l} u_{w}\frac{dw_{k}}{dt}=-w_{k}+b\underset{t_{sp}\left(k\right)}{\sum }\delta \left(t-t_{sp}\left(k\right)\right)+a\left(v_{k}-E_{L}\right), \end{array}$$

where *c*_*m*_ = 200 pF is the membrane capacitance, *v*_*k*_ is the voltage of neuron *k* and, whenever *v*_*k*_ > *v*_*peak*_ = −47.5 mV for inhibitory neurons and *v*_*k*_ > *v*_*peak*_ = −40.0 mV for excitatory at time *t*_*sp*_(*k*), *v*_*k*_ is reset to the resting voltage *v*_*reset*_ = −65 mV and fixed to that value for a refractory time *T*_*refr*_ = 5 ms. The voltage threshold *v*_*thr*_ is –50 mV. The leak term *g*_*L*_ had a fixed conductance of *g*_*L*_ = 10 nS and the leakage reversal *E*_*L*_ was of −65 mV for inhibitory and −63 for excitatory. The exponential term had a different strength for RS and FS cells, i.e., Δ = 2 mV (Δ = 0.5 mV) for excitatory (inhibitory) cells. Inhibitory neurons were modeled as fast spiking FS neurons with no adaptation (*a* = *b* = 0 for all inhibitory neurons) while excitatory regular spiking RS neurons had a lower level of excitability due to the presence of adaptation (while *b* varied in our simulations we fixed subthreshold adaptation *a* = 0 nS and *u*_*w*_ = 500 ms).

The synaptic current *I*_*syn*_ received by neuron *i* is the result of the spiking activity of all neurons *j* ∈ pre(*i*) pre-synaptic to neuron *i*. This current can be decomposed in the synaptic conductances evoked by excitatory E and inhibitory I pre-synaptic spikes

$$I_{syn}=G_{syn}^{e}\left(E_{e}-v_{k}\right)+G_{syn}^{i}\left(E_{i}-v_{k}\right),$$

Where *E*_*e*_ = 0 mV (*E*_*i*_ = −80 mV) is the excitatory (inhibitory) reversal potential. Excitatory synaptic conductances were modeled by a decaying exponential function that sharply increases by a fixed amount *Q*_*E*_ at each pre-synaptic spike, i.e.,:

$$G_{syn}^{e}\left(t\right)=Q_{e}\underset{exc.pre}{\sum }\Theta \left(t-t_{sp}^{e}\left(k\right)\right)\;e^{-\left(t-t_{sp}^{e}\left(k\right)\right)/u_{e}},$$

Where Θ is the Heaviside function, *u*_*e*_ = *u*_*i*_ = 5 ms is the characteristic decay time of excitatory and inhibitory synaptic conductances, and *Q*_*e*_ = 1.5 nS (*Q*_*i*_ = 5 nS) the excitatory (inhibitory) quantal conductance. Inhibitory synaptic conductances are modeled using the same equation with *e* → *i*. This network displays two different states according to the level of adaptation, *b* = 0 pA for asynchronous irregular states, and *b* = 60 pA for Up-Down states (see Zerlaut et al., 2018 for details).

### 4.2. Mean-field models

We considered a population model of a network of AdEx neurons, using a Master Equation formalism originally developed for balanced networks of integrate-and-fire neurons (El Boustani and Destexhe, 2009). This model was adapted to AdEx networks of RS and FS neurons (Zerlaut et al., 2018), and later modified to include adaptation (di Volo et al., 2019). The latter version is used here, which corresponds to the following equations using Einstein's index summation convention where sum signs are omitted and repeated indices are summed over:

(3) $$\begin{array}{l} T\frac{\partial \nu_{\mu }}{\partial t}=\left(F_{\mu }-\nu_{\mu }\right)+\frac{1}{2}c_{\lambda \eta }\frac{\partial^{2}F_{\mu }}{\partial \nu_{\lambda }\partial \nu_{\eta }} \end{array}$$

(4) $$\begin{array}{l} T\frac{\partial c_{\lambda \eta }}{\partial t}=\delta_{\lambda \eta }\frac{F_{\lambda }\left(1/T-F_{\eta }\right)}{N_{\lambda }}+\left(F_{\lambda }-\nu_{\lambda }\right)\left(F_{\eta }-\nu_{\eta }\right)\\ \;+\frac{\partial F_{\lambda }}{\partial \nu_{\mu }}c_{\eta \mu }+\frac{\partial F_{\eta }}{\partial \nu_{\mu }}c_{\lambda \mu }-2c_{\lambda \eta } \end{array}$$

(5) $$\begin{array}{l} \frac{\partial W}{\partial t}=-W/u_{w}+b\nu_{e}+a\left(\mu_{V}\left(\nu_{e},\nu_{i},W\right)-E_{L}\right)\;, \end{array}$$

where μ = {*e,i*} is the population index (excitatory or inhibitory), ν_μ_ the population firing rate and *c*_λη_ the covariance between populations λ and η. *W* is a population adaptation variable (di Volo et al., 2019). The function *F*_μ = {*e,i*}_ = *F*_μ = {*e,i*}_(ν_*e*_, ν_*i*_, *W*) is the transfer function which describes the firing rate of population μ as a function of excitatory and inhibitory inputs (with rates ν_*e*_ and ν_*i*_) and adaptation level *W*. These functions were estimated previously for RS and FS cells and in the presence of adaptation (di Volo et al., 2019).

At the first order, i.e., neglecting the dynamics of the covariance terms *c*_λη_, this model can be written simply as:

(6) $$\begin{array}{l} T\frac{d\nu_{\mu }}{dt}=\left(F_{\mu }-\nu_{\mu }\right)\;, \end{array}$$

Together with Equation (5). This system is equivalent to the well-known Wilson-Cowan model (Wilson and Cowan, 1972), with the specificity that the functions *F* need to be obtained according to the specific single neuron model under consideration. These functions were obtained previously for AdEx models of RS and FS cells (Zerlaut et al., 2018; di Volo et al., 2019) and the same are used here.

For a cortical volume modeled as two populations of excitatory and inhibitory neurons, the equations can be written as:

(7) $$\begin{array}{l} T\frac{d\nu_{e}}{dt}=F_{e}\left(\nu_{e}+\nu_{aff}+\nu_{drive},\nu_{i}\right)-\nu_{e} \end{array}$$

(8) $$\begin{array}{l} T\frac{d\nu_{i}}{dt}=F_{i}\left(\nu_{e}+\nu_{aff},\nu_{i}\right)-\nu_{i} \end{array}$$

(9) $$\begin{array}{l} \frac{dW}{dt}=-W/u_{w}+b\nu_{e}+a\left(\mu_{V}\left(\nu_{e},\nu_{i},W\right)-E_{L}\right), \end{array}$$

where ν_*aff*_ is the afferent thalamic input to the population of excitatory and inhibitory neurons and ν_*drive*_ is an external noisy drive simulated by an Ornstein-Uhlenbeck process. The function μ_*V*_ is the average membrane potential of the population and is given by

$$\mu_{V}=\frac{\mu_{Ge}E_{e}+\mu_{Gi}E+i+g_{L}E_{L}-W}{\mu_{Ge}+\mu_{Gi}+g_{L}},$$

where the mean excitatory conductance is μ_*Ge*_ = ν_*e*_*K*_*e*_*u*_*e*_*Q*_*e*_ and similarly for inhibition.

This system describes the population dynamics of a single region, and was shown to closely match the dynamics of the spiking network (di Volo et al., 2019).

### 4.3. Networks of mean-field models

Extending our previous work at the mesoscale (Chemla et al., 2019; di Volo et al., 2019) to model large brain regions, we define networks of mean-field models, representing interconnected brain regions (each described by a mean-field model). We considered interactions between cortical regions as excitatory, while inhibitory connections remain local to each region. The equations of such a network, expanding the two-population mean-field (Equation 7), are given by:

(10) $$\begin{array}{l} T\frac{d\nu_{e}\left(k\right)}{dt}=F_{e}\left[\nu_{e}^{input}\left(k\right)+\nu_{aff}\left(k\right),\nu_{i}\left(k\right)\right]-\nu_{e}\left(k\right)\\ T\frac{d\nu_{i}\left(k\right)}{dt}=F_{i}\left[\nu_{e}^{input}\left(k\right)+\nu_{aff}\left(k\right),\nu_{i}\left(k\right)\right]-\nu_{i}\left(k\right) \end{array}$$

(11) $$\begin{array}{l} \frac{dW\left(k\right)}{dt}=-W\left(k\right)/u_{w}+b\nu_{e}\left(k\right)\\ \;+a\left(\mu_{V}\left(\nu_{e}\left(k\right),\nu_{i}\left(k\right),W\left(k\right)\right)-E_{L}\right)\;, \end{array}$$

where ν_*e*_(*k*) and ν_*i*_(*k*) are the excitatory and inhibitory population firing rates at site *k*, respectively, *W*(*k*) the level of adapation of the population, and $\nu_{e}^{input}\left(k\right)$ is the excitatory synaptic input. The latter is given by:

(12) $$\begin{array}{l} \nu_{e}^{input}\left(k\right)=\nu_{drive}\left(k\right)+\underset{j}{\sum }C_{jk}\;\nu_{e}\left(j,t-||j-k||/v_{c}\right) \end{array}$$

where the sum runs over all nodes *j* sending excitatory connections to node *k*, and *C*_*jk*_ is the strength of the connection from *j* to *k* (and is equal to 1 for *j* = *k*). Note that ν_*e*_(*j, t* − ||*j* − *k*||/*v*_*c*_) is the activity of the excitatory population at node *k* at time *t* − ||*j* − *k*||/*v*_*c*_ to account for the delay of axonal propagation. Here, ||*j* − *k*|| is the distance between nodes *j* and *k* and *v*_*c*_ is the axonal propagation speed.

### 4.4. Spontaneous activity

The Phase-Lag Index (PLI) was computed for each pair of nodes, averaged over simulation time. The Hilbert transform is employed to extract the phase ψ(*t*) of the time series. From there, the PLI, given by

(13) $$\begin{array}{l} \text{PLI}\equiv |<sign\left(\psi_{i}\left(t\right)-\psi_{j}\left(t\right)\right)>|, \end{array}$$

is computed for nodes *i* and *j*, where < · > denotes averaging over time (Silva Pereira et al., 2017). One may note that the PLI takes values between 0 (random phase relations or perfect synchrony) and 1 (perfect phase locking). In this work we report the mean PLI over all time epochs for excitatory and inhibitory firing rates of each region pair for each adaptation value.

### 4.5. Parameter space exploration

A model such as the TVB-AdEx contains many parameters whose impact on the dynamics needs to be understood. Additionally, it is necessary to have reasonable, physiological ranges determined for them. As described above, most of them have were already fixed *via* biological or mathematical arguments, but there is still a subset of parameters whose impact needed to be studied to have a deeper and general understanding of the model. In Table 1, one can find the characteristics of the parameters chosen and the reason for their choice. For each parameter, 16 evenly spaced values were obtained inside the described range. Preliminary results allowed to reduce the explored parameter space to a total of 675,840 different configurations to be analyzed. Using High Performance Computing, the simulation of each parameter combination was parallelized. For each configuration, a seven second simulation was run and, afterwards, multiple features were extracted (mean and standard deviation of the excitatory and inhibitory firing rates, mean value of functional connectivity, etc.). By plotting the value of these features as a function of the parameter values, one can observe the influence of the latter on the model's dynamics (as is shown in Figure 6).

**Table 1:** Name, description, reason of choice, range and units of the parameters chosen for the parameter scan.

**Parameter**	**Description**	**Reason of choice**	**Range**	**Units**
*S*	Coupling strength between nodes. Has to be chosen phenomenologically.	Has to be chosen phenomenologically.	[0, 0.5]	Adimensional
*E*_*L,i*_	Leakage reversal potential of AdEx inhibitory neurons.	Resting membrane potential of a neuron might vary depending on external conditions.	[−80, −60]	mV
*E*_*L,e*_	Leakage reversal potential of AdEx excitatory neurons.	Resting membrane potential of a neuron might vary depending on external conditions.	[−80, −60]	mV
*T*	Timescale of the AdEx mean field model.	Has to be chosen phenomenologically.	[5, 40]	ms
*b*_*e*_	Adaptation strength of excitatory AdEx neurons.	Models the change in neuromodulation that induces transition between AI and UD.	[0, 120]	pA

### 4.6. Evoked activity

We computed the Perturbational Complexity Index (PCI) in response to a localized square wave stimulus, over the firing rates in a given brain region of a TVB-AdEx simulation, following the method proposed by Casali et al. (2013). This stimulus was applied by augmenting the firing rate of the excitatory population during the pulse. This is done for multiple trials with the same stimulus delivered to the same node at a random point in time and with different realizations of noise. The PCI is the ratio of two quantities: the Lempel-Ziv algorithmic complexity and the source entropy (Casali et al., 2013). To compute both quantities, firing rates ν(*t*) must be binarized to produce significance vectors *s*(*t*). First, the trials are aligned to stimulation time, considering only the 300 *ms* before and after stimulus onset. Then, each node's firing rate is re-scaled and mean and standard deviation given by pre-stimulus activity averaged over nodes. Afterwards, all pre-stimulus firing rates are randomized across time bins, this procedure being repeated 500 times. The threshold for significance *T* is then given by the one-tail percentile of the maximum absolute value over all repetitions within a series of 20 trials. For each trial of those 20 trials, we can then write *s*(*t*) = 1 whenever post-stimulus ν(*t*) > *T* and *s*(*t*) = 0 otherwise. For what follows, we concatenate all *s*(*t*) vectors from all simulation nodes into one single significance vector *S*(*t*) per trial.

The Lempel-Ziv complexity *LZ*(*S*) is the length of the “zipped” vector *S*(*t*), i.e., the number of possible binary “words” that make up the binary vector *S*(*t*). Briefly, *S*(*t*) is sectioned successively into consecutive words of between one and *N*_*t*_ characters where *N*_*t*_ is the total length of *S*(*t*). Scanning sequentially through all words, each new encountered word is added to a “dictionary,” and *LZ*(*S*) is the total number of words in the dictionary at the end of the procedure.

The spatial source entropy *H*(*S*) is given by:

(14) $$\begin{array}{l} H\left(S\right)=-p\left(S=0\right)\log_{2}\left(p\left(S=0\right)\right)-p\left(S=1\right)\log_{2}\left(p\left(S=1\right)\right), \end{array}$$

where log_2_ denotes the base-two logarithm.

The PCI can then be expressed as $PCI\left(S\right)=\frac{LZ\left(S\right)}{H\left(S\right)}$.

## Code availability

A python-based open-access code to run the present whole-brain model will be accessible online in the EBRAINS platform (https://ebrains.eu) as a companion to the publication of the present article. The scripts are also accessible on Github at https://gitlab.ebrains.eu/kancourt/tvb-adex-showcase3-git.

## Data availability statement

Publicly available datasets were analyzed in this study. This data can be found at: https://gitlab.ebrains.eu/kancourt/tvb-adex-showcase3-git.

## Author contributions

JG and LK wrote the models. JG, LK, BY, DD, KA, and DA characterized the models and ran simulations. T-AN, LK, BY, DD, KA, DA, and JG performed analyzes. JG, T-AN, VJ, and AD designed the work and wrote the manuscript. AD and VJ supervised the work. All authors contributed to the article and approved the submitted version.

## Supplementary material

Supplementary Figure 1 Inter-individual differences in model parameters predicted by personal connectomes from different healthy human subjects. High performance computing (HPC) grid search of parameters from a TVB-AdEx model constructed with a second unique human connectome (subject DH20120806 available from https://zenodo.org/record/3497545#.Y4YybezMK3I, compared to the TVB-AdEx model featured in the main text (subject QL20120814). Spectral features are extracted and differences between personalized TVB-AdEx models are shown in heatmaps. In particular, the two subject models show differing dependencies on connectivity strength and spike-frequency adaptation parameters, transitioning between wake- and sleep-like states at different values. Spectral profiles of simulated neural activity vary between subjects with distinct connectomes, with delta, theta, alpha power, and power law scaling showing differences in parameter space. With easily interchangeable individual connectomes from subjects in which behavioral and biological data are also available, TVB-AdEx models will help make testable personalized predictions regarding individual variation in transitions between conscious and unconscious brain states in healthy and pathological conditions.
